# Education for patients with rheumatoid arthritis in Latin America and the Caribbean

**DOI:** 10.1007/s10067-015-3014-y

**Published:** 2015-07-17

**Authors:** Vianna Khoury, Maria Kourilovitch, Loreto Massardo

**Affiliations:** Cátedra de Reumatología, Department of Medicine FCS, Pontificia Universidad Católica Madre y Maestra, Santiago, Dominican Republic; Department of Internal Medicine, School of Medicine, Cuenca State University, Cuenca, Ecuador; Department of Clinical Immunology and Rheumatology, School of Medicine, Pontificia Universidad Católica de Chile, Santiago, Chile

**Keywords:** Latin America, Patient education, Rheumatoid arthritis

## Abstract

Patient education is highly recommended in rheumatoid arthritis (RA) to support patient management. The challenge is to adhere to the recommendations for providing health education to RA patients in Latin American and the Caribbean (LAC) countries taking into account factors such as patient health illiteracy, lack of rheumatologists, and lack of resources including access to disease-modifying antirheumatic drugs (DMARDs). As existing educational material in regional languages is not readily available and inadequate, we propose developing a web-based educational program that would fulfill the requirements of most patients with RA across LAC countries with an emphasis on the correct and safe use of methotrexate.

Rheumatoid arthritis (RA) is a chronic inflammatory disease of the joints affecting 0.5–1 % of the adult population. RA produces pain, fatigue, and work incapacity, is potentially disabling, and shortens life expectancy. Much progress has been made in RA treatment in the last decades with methotrexate the cornerstone antirheumatic drug [1, 2]. Patient education is highly recommended in RA to support patient management. Guidelines for RA management say “Education for patients with RA should be provided since first medical encounter” [3]. Or recommend information to patients in the overarching principle “Treatment of RA patients must be based on a shared decision between the patient and the rheumatologist. Shared decision-making includes the need to inform the patient of the risks of RA and the benefits of reaching the targeted disease activity states, as well as the pros and cons of respective therapies. It also means two-way communication and joint or shared decision-making on the therapeutic target and management plan as well as support for the patient to develop personal preferences” [4]. Guidelines are endorsed by the Pan American League of Associations of Rheumatology (PANLAR) and all national rheumatology-affiliated societies and “Grupo Latino Americano de estudio De Artritis Reumatoide” (GLADAR) [5, 6]. Therefore, structured patient education should be available to all people with RA at initial diagnosis and on an ongoing basis, based on a formal, regular assessment of needs as the National Institute for Health and Care Excellence of the UK advocates (https://www.nice.org.uk/guidance/qs33). Implementing these recommendations and providing patient education in clinical practice in Latin America and the Caribbean is a challenge.

Some aspects that should be discussed in particular in the audio-visual material are listed here:

1. Adverse effects of medications in contrast to benefits.
2. Discuss diet and give dietary advice, taking into account other factors (e.g., obesity, hypertension, renal impairment); offer referral to a nutrition or dietician; also take into account hand/wrist incapacities to prepare food.
3. Encourage regular physical activity, taking into account pain, joint protection, and local facilities, and hydro gym.
4. Give advice and support on smoking cessation where appropriate.
5. Mobility, driving and using national transport system.
6. Getting help.
7. Finding positive activities.
8. Knitting? Embroidery? What there is the need for?
9. DAS28? What is it for?
10. How to ease morning stiffness.
11. Why should I care for my liver, my heart, my kidneys, and my bones?
12. Choosing shoes.
13. Dental hygiene.
14. My vaccines.
15. Avoiding people with TB.
16. When to seek help. Who is my contact if a drug adverse effect takes place?
17. What are biologics drugs?
18. What if I miss a dose of my medications?
19. What it means to be under immunosuppressive medication.
20. What medications can be combined?
21. Use of non steroidal anti-inflammatory drugs.
22. Marihuana for pain.
23. Sexuality and family planning.
24. Drinking wine and spirits.
25. Rights of patients with RA according to their country and health system.
26. Illness beliefs and integration of alternative and complementary medicine in RA therapy.
27. Work-related and lifestyle-related advice.

Inequities in health care are a reality in Latin America and the Caribbean (LAC). There are many problems to face regarding the actual standard of care of patients with RA in LAC, including few rheumatologists and very few allied health professionals working in RA [6]. Moreover, rheumatology is not part of the undergraduate curricula in many medical schools (results of a 2014 PANLAR survey, unpublished communication). For the majority of clinical rheumatologists, the workload is heavy, time assigned per visit is short, and waiting lists are long [5, 6]. Methotrexate in oral form is the disease-modifying antirheumatic drug (DMARD) available in most centers due to both low costs and its effectiveness in at least 50 to 75 % of patients. However, this may also depend on whether treatment was initiated within “the window of opportunity” whereas other DMARDs or biologics are difficult to access due to their expensive costs [7]. Understandably, in these working conditions, to fully apply treat to target strategies [4] is not possible. Health education for patients with RA and caregivers is not a priority, although rheumatologists would agree that it is necessary.

Therefore, it is relevant to develop a LAC health-educational program to help doctors in busy clinical practices to provide education to RA patients, taking into account difficulties attributable to health system organizations and working conditions, poverty, and other socioeconomic factors. Particularly, when a recent study in early RA patients from the GLADAR cohort showed that low/middle low socioeconomic status (SES) is associated with more active disease and worse functional capacity [8]. It is possible that a health education program based on this reality could positively impact the outcome of RA or health-related quality of life. Thus, patients would be able to make optimal and safe use of methotrexate [9] and other medications and therapies and adopt healthier conduct. In our opinion, patients from low/middle low SES, illiterate or semi-illiterate patients, patients living in rural or suburban conditions, or manual laborers who are at risk of losing their jobs are in most need of health education if they suffer from RA and possibly would benefit more. In LAC, the most social and economic support for RA patients is provided by the family, as pensions for disability, sick leave, or anticipated retirement are low and under the requirements for a severe disease, and most patients find little or no social support from the state or their health system coverage. Every effort to provide health educational material to all LAC patients with RA and their families must be done. Especially when considering that there are few rheumatologists and difficulties in accessing medical visits and free medications across LAC countries.

## Education measures for RA

Health education is a planned learning experience that influences the knowledge of the patient about his/her illness aiming to modify health habits/conduct in order to be able to collaborate in their therapy [10–12]. It utilizes a combination of learning methods, counseling, and techniques to modify conduct. It is an interactive process to help the individual to participate in their self-care and to make optimal use of all health resources. The relevance of patient education safety programs has been put forward by the Joint Commission on Accreditation of Healthcare Organizations (JCAHO) (http://www.jointcommission.org/speakup.aspx). As mentioned by Fox [13], the JCAHO requires patient education standards as a condition of accreditation, including requirements related to understandability and readability, ease of access to materials, and consideration of language differences and patient abilities. However, it is not clear what type of educational interventions is most effective in improving health status for patients with chronic diseases [14]. Education strategies can vary from the provision of information only to the use of cognitive-behavioral strategies. Formats include verbal, written, audio-visual, and interactive computer-based educational programs [13]. The objectives of patient education in RA are to improve patient outcomes as well as to obtain the best of the affordable therapies available. From 31 education trials in RA included in a meta-analysis, significant benefits of patient education at first follow-up are modest (5–12 %). The most important benefit was observed in functional disability; additionally, behavioral programs versus controls had better results than information only or counseling [15]. Verbal information given by physicians on how to take prescriptions or drug side effects is not understood, not recalled, or not remembered incorrectly affecting patient adherence [16] even though it is the most common format used in hospitals/clinics. Therefore, printed material and multimedia are likely better options.

Patient education is made up of knowledge, abilities and conducts, and psychological support. In RA, focus should be on obtaining and maintaining remission, prevention of relapses, and avoiding deformities and damage. If there is disability due to RA, how to adapt to this condition should be covered. Patients can learn the significance of adherence to therapies, attending regular controls, recognize possible complications on time, manage depression and anxiety symptoms, control co-morbidities, and avoid health risk conducts. Patients should be encouraged to maintain adequate body mass index and exercise, to follow vaccine programs, and to avoid infections. Patients should also adopt joint protection strategies [17]. Lack of awareness of health conducts directly affects patient’s response to treatment and outcomes or obtaining an acceptable health-related quality of life (http://www.uptodate.com/contents/arthritis-beyond-the-basics). Providing effective health education for RA patients has many difficulties. A recent study has shown that RA educational needs varies in relation to gender, personal characteristics, disease activity, and disease duration indicating that education be targeted more effectively. Correlations between educational needs and disease activity and function could enable identification of ‘intervention points’, which can be ideal opportunities for effective patient education [18].

A challenge often faced by LAC physicians when they approach patients with RA under their care is that they have health illiteracy or do not read, which both jeopardize patient compliance [19, 20] (http://www.health.gov/communication/). In LAC countries, patients often follow complementary and alternative medicine [21, 22] or magical cures that may greatly modify the therapeutic plan the rheumatologists consider more appropriate. An educational program should deal with doubts and help people with RA to take informed decisions considering risks and benefits [19]. When patients ask for information about RA, answers should be given in simple terms. However, this should not preclude offering the correct information particularly on methotrexate safe and correct use [19]. A respectful approximation of patient’s beliefs identifying the preoccupations, expectations, emotions, and interests of patients would allow the “coincidence of agendas of both patient and doctor” [23, 24] that would permit choosing the individualized health educational material. Therefore, we propose including courses on patient education as well physician-patient communication skills at a professional level of competence at PANLAR meetings as part of the continuing medical education in rheumatology.

There are suitable programs that could be applied to RA patients in groups such as the *Chronic Disease Self-Management Program of the Stanford University* [11] given in six workshops to 10–12 participants. People with RA that attended this workshop did not show any benefit in biomedical or lifestyle outcomes at 3 years, although there were sustained improvements in some illness beliefs. However, in a short period, the benefit was evident [12, 15, 25]. In 2006, training of people from ten Latin American countries in Panama was started but the effort did not continue and workshops were not repeated.

A review of web pages in Spanish shows sites providing information for patients and lay people on RA. The Medical Library of National Institute of Health from the US (http://www.nlm.nih.gov/medlineplus) and the American College of Rheumatology provide information for Spanish-speaking people with RA living in the USA explaining drug mechanisms of actions and adverse effects using commercial names in the USA. The National Institute of Arthritis and Musculoskeletal and Skin diseases web page offers little information (http://www.niams.nih.gov) whereas Wikipedia (http://es.wikipedia.org) offers an up-to-date text appropriate for those with biomedical knowledge. The *Sociedad Española de Reumatología* (www.ser.es) has developed a brochure with detailed explanations on pathogenesis, clinical features, and therapy; however, it is difficult for LAC patients to identify the drug names. Pharmaceutical companies publish educational material for patients that lack individualization and need revisions to assure the absence of bias. Common to all pages is the use of a minimum visual support, lack of audio, and no interactive web page in Spanish or Portuguese. In summary, there is a need for web-based material for patients adapted to the specific needs of LAC patients with RA and the diverse reality in LAC countries.

## Educational program for LAC RA patients: a proposal for a solution

The challenge is to adhere to the recommendations for providing health education to RA patients in LAC taking into account the previously explained difficulties and lack of resources. As existent material is not easily available and inadequate, we propose developing an educational program that would fulfill the requirements of most patients with RA across LAC countries. The proposal will need support from PANLAR, resources, and funding, but costs and details are not within the scope of this manuscript. We will briefly review the program we conjecture our patients would benefit from.

Our aim is that the RA patient educational material developed by PANLAR be offered to doctors and patients in a variety of formats the patient can choose from. The design will require short interventions with minimal contents explained clearly that could be given in each visit by the attending physician or under his/her direction and if possible, by health personnel and trained RA patients. This should be reinforced afterwards or at home.

The need to be scientifically valid is very importantly, and thus, every statement of the content will be based on evidence-based medicine or medical expert opinions when there is no evidence. As evidence changes, the program will be updated and reviewed annually.

We propose to develop a written and audio-visual material on a website based on plain local languages (in Spanish, including indigenous languages and Portuguese) that could be printed on one page in a letter case easily read (size 12), with headings and titles identifying the contents in a sequence that could be followed as needed. This material will be published online on the PANLAR educational website. As many patients, especially the elderly, do not have media access, they could be given printed material at the clinic visit, according to their needs. Videos should be posted on Youtube with frequently asked questions from a patient with an appointed doctor of about 5 min each. Rheumatologists, patients, linguist specialists, nurses, occupational therapists, physiotherapists, physiatrists, psychiatrists, psychologists, and education and multimedia experts will participate in the development of the material.

Additionally, patients will be directed to official ACR websites in Spanish and from the “Sociedad Española de Reumatología” and to EDUCAR, a website of PANLAR dedicated to continuing medical education in RA. Patient questions should be directed to the program by doctors or patients themselves to receive an expert response. A list of health providers containing rheumatology centers fulfilling local quality standards and qualified staff will be accessible from the web page of the educational site. A diffusion campaign to doctors and patient associations through email from PANLAR will be done. We are aware that the site should be found easily and be on the first two pages of Google.

## Content of the education program

*Knowledge*. Basic articular anatomy notions, inflammation notions, DMARDs mechanisms of action, and adverse effects with emphasis on the correct and safe use of methotrexate. What rheumatologists assess in a clinic visit such as articular indices and registries. Qualified/accredited health providers locally and at a national level. A list of available resources for RA and patient associations in LAC will also be included.

*Abilities and conducts*. Regular low-impact exercise combined with rest periods. Adaptation measures at work and in daily life. Promote adherence to therapy. Following vaccination schedule according to local regulations. Eating a healthy diet. How to proceed if an adverse effect appears.

*Psychological support including self-help and treatment for anxiety and depression when needed*. Communication strategies, support from family, and available social agencies. The table lists some aspects that should be discussed in particular in the audio-visual material.

## Evaluation of the education program

A survey will be sent out at 3 and 12 months to patient associations about readability, acceptance, and recall of the material and to rheumatologists about problems, questions, and suggestions. Also, a web page visit counts. This evaluation and revision will proceed annually.

In conclusion, there is an unmet need for patient health education in rheumatology in LAC, including patients with RA and their families. To reach more people with RA, information should be in various formats; written and audio-visual based on the web with an emphasis on the correct and safe use of methotrexate.
